# The Role of Vitamin D in Rare Diseases—A Clinical Review

**DOI:** 10.3390/biomedicines13030558

**Published:** 2025-02-22

**Authors:** Czesław Ducki, Marta Wojtkiewicz, Marcin Bartoszewicz, Piotr Fiedor

**Affiliations:** 1 Mazovian Specialized Health Center in Pruszków, Partyzantów 2/4, 05-802 Pruszków, Poland; 2 University of Economics and Human Sciences in Warsaw, Okopowa 59, 01-043 Warsaw, Poland; m.z.wojtkiewicz@gmail.com (M.W.); marcin.bartoszewicz@life.pl (M.B.); 3 Medical University of Warsaw, Żwirki i Wigury 61, 02-091 Warsaw, Poland

**Keywords:** rare diseases, vitamin D status, quality of life, drug interactions, immunoregulation, clinical practice guidelines, precision medicine

## Abstract

**Background/Objectives:** Patients suffering from rare diseases are particularly vulnerable to vitamin D deficiency. The role of vitamin D status in rare disease management remains insufficiently investigated and employed in routine clinical practice. **Methods:** This review analyses current data on vitamin D status in selected rare diseases of organs involved in vitamin D metabolism: skin (epidermolysis bullosa, morphea), liver (autoimmune hepatitis, primary biliary cholangitis, primary sclerosing cholangitis), kidney (Alport syndrome, Fabry disease), and cystic fibrosis as a model of a systemic rare disease. Additionally, this review critically examines potential drug–vitamin D interactions in the context of rare disease patient polypharmacy. **Results:** Evidence suggests that vitamin D deficiency is prevalent in rare disease patient populations, often at once exacerbating and being simultaneously exacerbated by the underlying condition. Vitamin D deficiency correlates with worse clinical outcomes and lower quality of life across the examined diseases. Immunoregulatory properties of vitamin D appear relevant for rare diseases with autoimmune components. **Conclusions:** An urgent need for developing disease-specific clinical practice guidelines, implementing routine vitamin D monitoring in rare disease patient care, and introducing tailored supplementation under the principles of precision medicine is emphasized.

## 1. Introduction

The number of rare diseases (RDs), defined in the European Union (EU) as having a prevalence of less than 5 per 10,000 persons [1], is currently estimated to surpass 10,000 [2]. The cumulative population prevalence of RDs is estimated to be between 3.5% and 5.9%, translating to between 263 and 446 million patients afflicted globally and an upper boundary estimate of 36 million in just the EU [3]. RD patient medical care is challenging due to the need for (systemic) symptom alleviation and comorbidity (including infection) management. This sometimes represents the only strategy of medical intervention, as a vast majority (around 94% to 95%) of RDs lack approved prognosis-changing treatment [4]. Trend analysis points to patients with rarer diseases being prescribed more medications and concurrently medicinal products not as commonly used in the general patient population. This additionally confounds existing medication recommendation systems—big data learning models—which are tools for supporting clinician therapeutic decisions, leading to their lower accuracy in the case of RD patients [5]. RD patients are, therefore, subject to polypharmacy and at an increased risk of harm owing to potentially inaccurate clinical decisions, which increases the risk of the occurrence of drug-drug interactions [6].

The 1α,25-hydroxylated, active hormonal form of vitamin D_3_—calcitriol (1,25(OH)_2_D_3_) exerts its activity primarily via interaction with the vitamin D receptor (VDR). This steroid hormone nuclear receptor family member undergoes heterodimerization with retinoid X receptors (RXR) following active vitamin D binding. The receptor-ligand complex alters the target gene expression of a wide array of unique loci, leading to chromatin compaction changes and gene transcription up- or downregulation [7]. The wide range of biological functions exerted by vitamin D includes calcium and phosphate metabolism regulation, modulation of cellular proliferation and differentiation, innate and adaptive immune regulation, and an anti-oxidative effect [8,9]. This pleiotropic role of vitamin D is reflected in the expression of VDRs in cells of the immune system (including monocytes, macrophages, dendritic cells, and T-lymphocyte populations), osteoprogenitor cells, osteoblasts, osteocytes, chondrocytes, the intestinal mucosa, pancreatic beta cells, kidney tubular, bronchial, thyroid, parathyroid, prostate gland secretory and skin epithelial cells, and germline cells [10]. The prohormone form of vitamin D originates from dietary intake (including the alternative and functional equivalent form of vitamin D_2_) or synthesis from 7-dehydrocholesterol in the skin under exposure to UVB light wavelengths. It is transported in the blood bound to the carrier vitamin D binding protein (DBP), to then be hydroxylated by CYP family hydroxylases in the 25- and α1- positions, principally in the liver (by CYP2R1, 3A4, 27A1) and kidney (by CYP27B1) respectively, or in local tissue hydroxylation systems [11,12]. Vitamin D metabolism and its wide range of physiological functions have been subject to literature discussion in multiple clinical studies and reviews.

The RD patients’ inherent additional burden of their underlying condition makes them even more vulnerable to vitamin D deficiency relative to the already-deficient general population [13]. Apart from a general impairment of nutritional intake (including fat and fat-soluble vitamin malabsorption) and status (including fat stores depletion), RD patients may be prone to lower exposure to sunlight (due to impairment of mobility, dressings covering a significant area of the skin [14] or strict sun exposure avoidance), impaired vitamin D hydroxylation in systemic RDs such as cystic fibrosis [15], or locally impaired cytochrome function in affected organs in RDs of the liver [16], the effects of prescribed medications such as glucocorticoids [17], or non-adherence to supplementation regimens.

This paper aims to provide a summary of the existing clinical consensus and a broad overview of the literature on vitamin D status significance in a selection of RDs—RDs of organs involved in vitamin D metabolism—the skin (epidermolysis bullosa—EB, ORPHA:303, ORPHA:304, ORPHA:305; morphea ORPHA:90289), liver (autoimmune hepatitis—AIH, ORPHA:2137; primary biliary cholangitis—PBC, ORPHA:186; primary sclerosing cholangitis—PSC, ORPHA:171), and kidney (Alport syndrome, ORPHA:63; Fabry disease, ORPHA:324). Cystic fibrosis, as representative of a systemic RD model of challenges of vitamin D dietary intake, absorption, and metabolism, including decreased exposure to sunlight, the effect of medication, and altered hydroxylation, is examined (cystic fibrosis—CF, ORPHA:586).

Additionally, the current state of knowledge regarding vitamin D’s known range of immunoregulatory function is briefly reported in relation to the immune dysregulation in RDs. Finally, the literature on potential drug-drug interactions with vitamin D supplement medicinal products is critically examined in relation to RD patient polypharmacy.

A compendium of recent clinical trials (without posted results) in these RDs with vitamin D as either the intervention or outcome measure is outlined below in Table 1. CF trials with results posted are presented separately in the relevant section.

## 2. Review Methodology

The last decade (2014–2024) was initially set as a relevant timeframe for search for articles on the subject matter to be identified in the PubMed (http://www.ncbi.nlm.nih.gov/pubmed/ First access on 1 September 2024) and Google Scholar (https://scholar.google.com/ First access on 1 September 2024) databases. Keywords were used to streamline the search towards the scope of the review: rare diseases (and terms for specific RDs of the skin, kidneys, liver, and cystic fibrosis, as per the list of RDs selected for the scope of review) AND vitamin D, vitamin D supplementation, vitamin D status, quality of life, and separately vitamin D AND drug interactions, immunoregulation, autoimmunity. Forty-two references were selected according to this timeframe and specified keywords. Priority was given to clinical studies (cohort, case–control, and observational). Additional articles were identified by reviewing reference lists, and the timeframe was extended to 20 years. Relevant publications on animal models and molecular mechanisms (no timeframe was selected here) were also included where applicable. A systematic study selection approach was not implemented due to an insufficient number of RCT studies for all diseases and wide differences in protocols. Data were extracted based on their relevance to the topic. Studies not available in English were excluded. The database of clinical trials (https://clinicaltrials.gov/ First access on 1 September 2024) was searched for trials with vitamin D as the intervention in the broad category of rare diseases and separately for each of the selected rare diseases, with a 20-year timeframe.

## 3. RDs of the Skin—EB, Morphea

Epidermolysis bullosa (EB) refers jointly to a group of inherited skin blistering disorders, with a wide range of causal gene mutations (including epidermal keratins or collagen type VII) causing mechanical fragility of the tissue. While EB severely negatively impacts patient quality of life (QoL), various dressings, including biological Advanced Therapy Medicinal Products (ATMPs), are used in patient care [18].

Recessive dystrophic EB (RDEB) and junctional EB (JEB) are particularly severe forms of the disease associated with chronic wounds and secondary systemic involvement [19]. In these generalized forms of EB, impairment of bone status is an associated complication, with both high disease severity scores in the Birmingham scale and low 25(OH)D serum levels showing an association with low bone mass. Rodari et al. found a significant correlation between areal bone mineral density and the two aforementioned clinical parameters in an observational group of 20 children with EB [20]. Yuniati et al. reported on a case of rickets and osteomalacia in a 21-year-old female with severe EB presentation. The measured 25(OH)D level marked severe deficiency at 8.6 ng/mL. The patient was prescribed 5000 IU vitamin D once daily and ceramide-based moisturizing cream for 6 months, leading to fewer skin blistering lesions and no new lesions appearing in the follow-up period [21].

Patients with EB are prone to vitamin D deficiencies from their early years due to reduced mobility and exposure to natural UVB light, impaired nutrition, dressings covering significant skin areas, low palatability of supplements, cost of supplementation, and a lack of adherence to supplementation regimens. In a pediatric-population study on 24 patients with RDEB by Yerlett et al., 54% had vitamin D deficiency or insufficiency, which was resolved by starting or increasing supplementation in 69% of those children. In the remaining four cases, non-adherence persisted, and so did the deficiency. The required dose to maintain sufficient vitamin D status was found to increase with age and grow up to three times the norm for the general population.

In a cohort study on 200 patients by Reimet et al., vitamin D deficiency was identified in 67% of RDEB and 76% of JEB patients, alongside a high prevalence of zinc (55% and 94%) and selenium (32% and 75%) deficiencies, with a significant correlation of low weight in RDEB with low serum levels of zinc and vitamin D. These nutritional deficiencies were already present in the second year of life of pediatric patients and emerged despite adherence to recommended supplementation.

A recently published initiative undertaken as of January 2024 by DEBRA International (a leading EB patient advocacy and support network) issued proposed clinical practice guidelines (CPG) for EB, initiating the long-term process of CPG consensus development, focusing on the neonatal population. DEBRA International experts jointly recommend the monitoring of iron, zinc, vitamin K, and vitamin D levels for patients with large degloving wounds. They urge the consideration of prophylactic multivitamin and mineral supplementation while underlining that vitamin requirements in children with EB can be 150–200% of the recommended daily intake for the general population [22]. EB, therefore, functions as a proxy model for the challenges in vitamin D status relevant to skin RDs and as a model for expert action to be undertaken on this topic.

Morphea (localized scleroderma) is an autoimmune RD characterized by inflammation and sclerosis of the skin and underlying tissue, with relapses of flare-ups leading to permanent damage (including tissue and pigmentation loss). The disease lacks the autoantibodies specific to systemic sclerosis despite having similar histology. A multifactorial etiology involving dysregulated immune and fibrotic pathways is currently postulated [23].

Morphea is of interest in the context of vitamin D, as some studies indicate that topical vitamin D analogs (calcipotriol) are a treatment option, resulting in a significant reduction of erythema, dyspigmentation, telangiectasis, and induration, whether used in addition to topical steroids or as a monotherapy. Certain studies supplement this medicinal product therapy with phototherapy. A need persists for randomized, placebo-controlled trials [24]. On the other hand, a case-control study by Yildirim et al. showed no relationship between VDR polymorphisms, vitamin D serum levels, disease subtype, age of onset, and responsiveness to treatment in morphea [25].

## 4. RDs of the Liver—PBC, PSC, AIH

The liver plays a key role in regulating vitamin D activity in the body, as it is the location of cholecalciferol being hydroxylated in the 25th position to the prohormone calcifediol, which then undergoes further enzymatic modification in the kidneys or local tissue systems. Calcifediol (25(OH)D) is the main form of vitamin D measured when assessing its serum levels (its concentration is in the ng/mL range, as opposed to pg/mL in the case of 1,25(OH)D). It can be seen as a derivative of liver function. Thus, liver pathologies often cause vitamin D deficiency, whereas such a deficiency can further aggravate the effects of the primary disease. Three RDs in which a relationship to vitamin D levels has been shown are primary biliary cirrhosis (PBC), primary sclerosing cholangitis (PSC), and autoimmune hepatitis (AIH) [26].

PBC is anRD in which the small bile ducts of the liver undergo progressive destruction, leading to bile accumulation, cholestasis, then inflammation, and, ultimately, cirrhosis. In PBC, vitamin D levels below 50 μmol/L are associated with a higher risk of cirrhosis and heightened mortality. Using a multivariable Mendelian randomization analysis of over 12,000 patients, vitamin D deficiency has been identified as an independent causal factor for PBC [27]. Another study confirmed a significantly higher frequency of liver transplants and liver-related mortality in patients with vitamin D deficiency. In this study, an increase in serum vitamin D levels by 1 μmol/L was associated with a 3% decrease in liver-related events [28]. Studies have also shown increased liver enzymes, decreased albumin, and lower histological scores for deficient patients. This has been attributed to the role of vitamin D in decreasing collagen I and III expression and increasing metalloproteinase activity while also having an immunomodulating role, decreasing pro-inflammatory cytokine release. Additionally, vitamin D diminishes the activation of hepatic stellate cells and consequent fibrosis. Furthermore, all studies point to vitamin D deficiency causing a decreased response to ursodeoxycholic acid, which is a drug of first choice in PBC treatment [29]. This may be due to a synergic effect of both substances in immunomodulation, which is absent in deficient patients. However, no guidelines exist regarding the supplementation of vitamin D in PBC, and as such, it is difficult to study the role of supplementation in disease progression in large population samples.

In PSC, both the small internal and extrahepatic biliary ducts are destroyed through mechanisms that are thought to be autoimmune. The role of vitamin D in PSC is of note, as it has a suppressive effect on T lymphocytes, which accumulate around the sclerotic ducts and exacerbate inflammation. Similarly to PBC, vitamin D deficiency correlates with a higher incidence of liver-related episodes, the need for transplants, and ultimately mortality, and one study points to a severe deficiency of under 25 μmol/L as being an independently correlated factor. Notably, patients who were persistently deficient over all measured time points had a twice higher risk of poor clinical liver outcomes. However, vitamin D deficiency, when adjusted for confounding factors, was found to not affect hepatobiliary malignancy development [30]. Similarly to PBC, no guidelines regarding supplementation currently exist.

AIH is an RD presenting as hepatic inflammation, which can ultimately result in liver failure and cirrhosis. In AIH, vitamin D exerts its positive effects through genomic and non-genomic mechanisms. The genomic mechanism consists of increasing intracellular Ca^2+^ and DNA polymerase activity, as well as upregulating the expression of CYP450 enzymes, aiding detoxification [31]. Vitamin D also decreases Toll-like receptor (TLR) and increases CTLA-4 and IL-10 expression, both of which inhibit cytotoxic T-lymphocyte proliferation and favor the regulatory T-cell population [32]. Conversely, a non-genomic effect on AIH is by inhibiting MHCII presentation on immune cells, which has a positive influence on decreasing inflammation. Additionally, vitamin D works to decrease INF-gamma production, protects hepatocytes from oxidative stress by reducing reactive oxygen species production, and enhances intracellular glutathione levels, acting as an additional barrier from oxidation [33]. However, as in the case of the previous two diseases, there is neither a proven link between increasing vitamin D serum levels and a decreased prevalence of the disease nor any guidelines for the supplementation of vitamin D in AIH.

In rare hepatic diseases, low vitamin D serum levels are associated with worse prognosis and higher mortality due to its immunomodulating and antifibrotic properties. However, a cause-effect relationship has not yet been proven, and as such, it remains difficult to ascertain whether deficiencies are a result of the ongoing disease process or whether they precede and contribute to the development of the illness. Furthermore, guidelines are lacking when it comes to the mode, doses, and frequency of vitamin D supplementation.

## 5. RDs of the Kidneys—Alport Syndrome, Fabry Disease

The kidney plays a crucial role in the last stage of synthesis of the biologically active, hormonal form of vitamin D by hydroxylating 25-hydroxycholecalciefrol in the 1α position to 1α,25-dihydroxycholecalciferol by the action of the enzyme 1-α-hydroxylase, which is present in the proximal tubules. From there, the hormone is transported to target sites in the organism through the bloodstream. Additionally, the enzyme 24-hydroxylase is also active in the kidneys and acts oppositely to reduce the amount of active vitamin D_3_ in the case of an excess [34]. Thus, if the function of the kidneys is impaired, this can affect vitamin D levels and often manifests in the form of disbalances in calcium–phosphate homeostasis [35].

One kidney RD which has been linked to vitamin D deficiency is Alport syndrome. This genetic condition involves a mutation in the gene encoding collagen IV, which in turn causes its deposition in the glomerular membrane, which is then unable to serve its filtrating function adequately, ultimately leading to renal failure [36]. This dysfunction of the kidney also affects vitamin D and electrolyte levels. The vitamin is hydroxylated less efficiently as the disease progresses, and phosphates accumulate due to ineffective excretion, leading to a rise in parathormone levels and higher bone turnover in a compensatory mechanism to increase calcium availability. Thus, Alport syndrome can have severe manifestations linked to the skeletal system, with one study reporting extreme maxillofacial complications of the disease [37]. As such, paricalcitol, an active vitamin D analog, has been recommended for the treatment of Alport syndrome (and chronic kidney diseases in general), as it has been shown to reduce mortality, inhibiting renin release and thus exerting positive effects on the cardiovascular system [38].

Another RD with a renal manifestation is Fabry disease, which belongs to the family of lysosomal storage diseases and is inherited in an X-linked manner. In Fabry disease, there is a deficiency of alpha-galactosidase, which is responsible for the degradation of sphingolipids. This causes their accumulation in cells and, in the case of the renal system, causes proteinuria, progressive loss of function, and, ultimately, kidney failure, which is often a cause of death [39]. Among Fabry disease patients, vitamin D deficiency is common and has been estimated to stand at 73%. This is exacerbated by the fact that symptoms often worsen with sunlight exposure, leading patients to avoid it. In deficient patients, proteinuria levels are higher, as are complications related to other systems. It has been suggested that vitamin D plays a direct causal role in regulating kidney function and the glomerular filtration rate. In a study of diabetic mice, knocking out the vitamin D receptor led to a thickening of the glomerular membrane and increased proteinuria [40]. Supplementation of vitamin D in patients with diabetic nephropathy caused a decrease in residual albuminuria [41]. Additionally, low vitamin D levels have also been associated with poor cardiovascular course in Fabry disease, the heart being another organ affected by the disease [42]. Therefore, while no recommendations exist regarding vitamin D intake in Fabry disease, it can be speculated that dietary supplementation may be beneficial to patients.

## 6. Cystic Fibrosis

Cystic fibrosis (CF) is a disease affecting the chloride transmembrane transport channel—cystic fibrosis transmembrane conductance regulator (CFTR), present in the epithelial cells of many vital organs. Vitamin D deficiency is present in up to 90% of patients suffering from CF, despite supplementation, and is attributed to the poor absorption of fat due to pancreatic exocrine insufficiency, limited exposure to sunlight, and alterations in the hydroxylation process in the body [43]. Even with pancreatic enzyme replacement therapy, deficiency can often not be mitigated, and sunlight exposure is difficult due to photosensitivity from antibiotics, which are taken regularly to prevent or treat infection [44]. Other medicines in CF treatment regimens increase the metabolism of vitamin D, resulting in faster elimination [45].

A crucial mechanism through which vitamin D can play a positive role in CF is by increasing the expression of cathelicidin, an antimicrobial peptide that decreases the frequency and severity of bacterial pneumonia, a common occurrence in CF patients [46]. Conversely, bacterial infections can also increase the activity of α1-hydroxylase and, thus, the level of serum vitamin D concentrations by activating the TLR on macrophages [47]. Vitamin D also has a positive influence on lung function and is associated with increased FEV1 and FVC. This may be due to reduced inflammation, less frequent bacterial infections, and increased airway remodeling after pulmonary exacerbations [48]. Additionally, vitamin D deficiency has been associated with a poorer Lung Clearance Index, a measure of tidal lung volume, which is especially effective for measuring lung function in children and adolescents [49].

Organ systems other than the lungs also suffer from decreased vitamin D levels in patients with CF. Bone density is lower in these patients, leading to a higher prevalence of osteoporosis and fractures, which have been estimated at around 20%, due to secondary hyperparathyroidism and increased calcium resorption from bones [50]. Vitamin D deficiency has also been linked to a higher incidence of CF-related diabetes, while higher vitamin D levels have been shown to decrease the risk of this comorbidity [51].

In CF, comprehensive guidelines exist concerning both the target levels of vitamin D and the manner of supplementation, considering the age of patients. The Cystic Fibrosis Foundation recommends that patients with CF have a serum vitamin D level of at least 30 μg/L and that this be measured annually, preferably in winter. Vitamin D should be supplemented in the form of cholecalciferol, vitamin D3, at an initial dose of 800–1000 IU a day, which can be increased to up to 10,000 IU daily. Additionally, the use of UV lamps is suggested but is not part of official recommendations and is up to individual clinical decisions [52]. However, despite these guidelines being in place, deficiency, even at severe levels of <20 μg/L, is still widespread among CF patients. This has been linked to poor adherence to supplementation regimens, poor absorption of the supplemented vitamin from the intestinal tract, and avoiding the outdoors [53]. On the other hand, vitamin D intoxication is extremely rare, despite overdosage occurring in about 5% of CF patients, but has been reported in some patients where the vitamin was incorrectly supplemented. One such case describes a 4-year-old girl, presenting with polyuria, polydipsia, fatigue, and weight loss, who had been consuming 8,000,000 IU daily due to a dilution error by a pharmacist. She was effectively treated with loop diuretics, bisphosphonates, and hyperhydration, but vitamin D levels remained elevated for over 2 months. As such, clinicians must be aware of the common symptoms of vitamin D overdose and be alert to this possible complication in CF patients [54].

Table 2 below presents clinical trials in CF in the vitamin D category (intervention and/or outcome) with posted results.

## 7. Immunoregulatory Role of Vitamin D

Many RDs, including the ones discussed in this article, have an autoimmunity component. The role of vitamin D in immunoregulation and prevention of autoimmunisation is a crucial topic for understanding the clinical translation of vitamin D status.

Vitamin D promotes the proper functioning of immune cells, as VDR signaling affects multiple cell lineages, including monocytes, dendritic cells, and T-lymphocytes. The hydroxylase CYP27B1 expressed by immune cells allows for the local conversion of vitamin D to the active hormonal form. The effects of vitamin D include skewing T-cells towards Th-2 polarization and thus humoral immunity, inhibiting antigen presentation and inflammatory cytokine production (including TNFα, IL-2, IL-17 expression), and promoting Treg activity, enhancing NKT functioning, the generation of antimicrobial peptides (cathelicidin), and anti-oxidation. Deficiencies of vitamin D are routinely present in patients with multiple sclerosis (MS), diabetes mellitus type 1 (DM1), and systemic lupus erythematosus (SLE). Overall, vitamin D can be said to promote innate immunity while inhibiting adaptive immunity and promoting a tolerance response.

A review by Dipasquale et al. analyzed evidence of the role of vitamin D levels in autoimmune diseases (also common diseases including thyropathies), concluding a need for further establishment of whether the role of vitamin D levels is causative or consequential in prevention. In a retrospective cohort study by Tao et al., patients with low vitamin D levels had an increased inflammatory response, oxidative stress, and a reduction in T cell subsets—25(OH)D levels were found to correlate negatively with pro-inflammatory factors (CRP, TNF, IL-6) and oxidative stress factor (malondialdehyde) and positively with CD3+ and CD4+ counts, superoxide dismutase, and total antioxidant capacity. The deficiency negatively affected liver function. The authors confirmed the consensus that vitamin D should be provided to all newborns during their first year of life, and afterward, the vitamin D supplementation regimen should be tailored to the presence of risk and specific disease [55,56]. Some studies have even pointed to the additive effects of glucocorticoids and vitamin D on inhibiting human lymphocyte and monocyte proliferation [57].

## 8. Drug-Vitamin D Interactions

The topic of drug–vitamin D interactions has so far been insufficiently investigated, with the results of many clinical studies being statistically inconclusive, contradicting comparable studies, and attempts at obtaining repeated results leading to different conclusions. The potential of such interactions, whether it be on the level of metabolism or adverse effects, is necessary for healthcare professionals to consider, particularly in RD patients, due to polypharmacy. This is despite the general public presentation of vitamin D supplementation as connected exclusively to health benefits, independently of the patient’s comorbidities.

Three biological mechanisms have been identified as sources of the potential drug interactions with vitamin D—alteration of CYP hydroxylase status, fat metabolism, or calcium-phosphate metabolism.

The role of CYP hydroxylases, which, as mentioned previously, convert successive forms of vitamin D to the main 1,25(OH)_2_D serum-circulating form in drug metabolism (particularly phase I biotransformation), is well established. Particularly of note is CYP3A4, which is involved in the hydroxylation of vitamin D in the liver and, based on in-vitro evidence, in the metabolism of approximately half of all medicinal products. CYP3A4 exhibits activity in the hepatocytes but also in the intestinal mucosa. Interactions may result between drugs requiring CYP3A4 activation or de-activation and those inhibiting or increasing the hydroxylase activity. Based on the location of CYP3A4 expression, it is stipulated that interactions may vary based on the route of drug administration and the source of vitamin D, whether that be dietary supplementation or UVB exposure (while being potentially less significant for IV drug administration and natural synthesis of vitamin D precursors in the skin). VDR activation additionally causes the upregulation of CYP3A4, as the expressed gene has an appropriate response element. The absorption of oral vitamin D supplements may be impaired by drugs altering fat absorption in the gastrointestinal tract. Finally, hypercalcemia is reported as a possible side-effect in older people under regimens of supplementation and calcium-sparing or calcium-containing medications.

Evidence from a metanalysis of clinical studies by Robien et al. points to no significant effect of bile acid sequestrants (colestipol, cholestyramine), epileptic drugs (this is ground for further research as studies usually accommodated ambulatory patients with varying regimens, while animal studies showed synergetic effects, as the regulatory (anti-oxidative, neuroprotective effect—affecting neuromodulator release) function of vitamin D was shown to enhance lamotrigine effectiveness in rat models [58]), glucocorticosteroids, ranitidine (as opposed to cimetidine, a CYP inhibitor), or immunosuppressants (tacrolimus, ciclosporin) on vitamin D status. Despite that, as immunosuppressants and glucocorticosteroids have well-known side effects relating to bone metabolism, osteopenia, and osteoporosis, it may be prudent to still monitor vitamin D serum concentrations.

The effect of orlistat—a lipase inhibitor is uncertain, as in the presented study, sera concentrations of 25(OH)D also decreased in the control group, showing a possibility that a decrease in dietary fat intake may have been the cause for vitamin D status change in both study arms. A potential interaction was shown between statins metabolized by CYP3A4 (atorvastatin, lovastatin, simvastatin)—where vitamin D supplementation (800 IU/d) caused a significant decrease in drug sera concentration, HAART drugs—again strongly affecting CYP3A4, with reporting of statistically lower serum levels of 25(OH)D, and of rifampin and isoniazid, anti-tuberculosis (TB) drugs, a CYP3A4 inducer and inhibitor respectively, with an unclear effect depending on the study. This is crucial as vitamin D deficiency has been connected to TB infection or reactivation susceptibility.

Reports have been made of hypercalcemia during simultaneous vitamin D and thiazide intake; however, it must be underlined that it occurred in specific cases, with joint regimens of vitamin D supplementation and additional calcium intake, in older patients (e.g., a 78-year-old woman taking vitamin D_2_ (50,000 IU/day), calcium carbonate (1.5 g elemental calcium/day) and hydrochlorothiazide (25 mg/day)) and were reversible, leading to a general conclusion that no significant alterations are a typical result of thiazide treatment. A review by Wakeman analyzed the impact of drug groups on vitamin D status, pointing to a negative association of 25(OH)D concentrations with the administration of metformin, calcium channel blockers (verapamil and diltiazem, being CYP3A4 inhibitors), heparin, SSRIs, and sulphonamides, while aggregating studies reporting mixed findings in the case of ACE inhibitors, loop and potassium-sparing diuretics, vitamin K antagonists, platelet aggregation inhibitors, digoxin, and benzodiazepines, with contradictory results in clinical analysis [59].

In animal and cell models, vitamin D was shown to support the efficacy of drugs such as immunosuppressants, anti-epileptics, and memantine in Alzheimer’s and prevent adverse reactions—e.g., the nephrotoxicity of immunosuppressants or gentamicin [60].

In general, no interactions have been identified significantly between cancer therapy drugs and vitamin D; however, a metanalysis by Kennedy et al. once again pointed to the need for consideration of thiazide therapy and vitamin D supplementation in older people due to reports of hypercalcemia [61].

## 9. Discussion

The comprehensive review emphasizes the pleiotropic role of vitamin D in RDs, highlighting both the significance of supplementation and the challenges in maintaining optimal vitamin D status in RD patients. The literature-based evidence presented demonstrates that vitamin D deficiency is prevalent in RD populations, often at once exacerbating and being simultaneously exacerbated by the underlying condition.

The examined RDs of the skin (EB, morphea), liver (PBC, PSC, AIH), kidneys (Alport syndrome, Fabry disease), and the systemic condition of CF all show associations with vitamin D status. In these conditions, vitamin D deficiency correlates with worse clinical outcomes and a lower QoL.

The immunoregulatory properties of vitamin D appear relevant—with modulation of the inflammatory response, lymphocyte population proportion, and oxidative stress, given the autoimmune component present in many RDs, and the drug-vitamin D interactions are essential to consider as RD patients are subject to polypharmacy.

However, several critical gaps in current knowledge and, therefore, in the implementation of clinical practice emerge from this review.

While associations between vitamin D deficiency and disease severity are well-documented, the causative relationship remains unclear in many RDs. Except for CF and, up until recently, EB, most RDs lack standardized guidelines for vitamin D supplementation and monitoring. Finally, the complex interplay between vitamin D and various medications commonly prescribed requires further investigation, particularly given the lack of significant conclusions in a number of studies.

There exists an urgent need for developing disease-specific vitamin D supplementation guidelines for RDs, large-scale, randomized, controlled cross-border clinical trials to establish optimal supplementation regimens and to analyze drug-vitamin D interactions in the context of RD polypharmacy. This should ultimately lead to the implementation of routine vitamin D status control in RD patient care, which is appropriate for the patient under the principles of *precision medicine.*

As RD clinical management continues to develop, maintaining optimal vitamin D status should be considered an integral component of comprehensive patient care.

## 10. Conclusions

A review and analysis of the literature strongly indicate that vitamin D plays a key role in the analysis of rare diseases. Additionally, supplementation of vitamin D directly influences serum levels and indicates a key role in immune response and protection from pathological activity in immune auto-aggression diseases. Data do not strongly suggest an interaction between medicinal products in standard schemes of treatment and vitamin D supplementation. The QoL improves significantly compared to patients without supplementation.

## Figures and Tables

**Table 1:** Clinical trials in discussed RDs in the vitamin D category (intervention and/or outcome)—no results posted.

Title	Sponsor	Years	RD
Vitamin D Status and Bone Metabolism Status in Children With Congenital Epidermolysis Bullosa	NCT05141838 National Medical Research Centre for Children’s Health, Russian Federation	2020–2023	EB
Molecular Effects of Topical Calcipotriene on Morphea	NCT02411643 Northwestern University	2015–2016	Morphea
Immunomodulating Effects of Supplementation With 25-OH Vitamin D (SCLERODERMA)	NCT04822038 Coordinación de Investigación en Salud, Mexico	2017–2019	Scleroderma
Empirical Comparative Study of Variation Blood Level Antibody Vitamin D at Scleroderma (SSc) Patients Compared Healthy Peoples (SSc)	NCT01553890 Meir Medical Center	2012–2013	Scleroderma
The Effectiveness of Combining Ursodeoxycholic Acid With Vitamin D in Treating Patients With Primary Biliary Cholangitis	NCT06309589 Yilihamu·Abilitifu, People’s Hospital of Xinjiang Uygur Autonomous Region	2021–2023	PBC
Comparison of Absorption of Vitamin D in Cystic Fibrosis	NCT01880346 Emory University	2013–2015	CF
Increased Vitamin D Reduces Pulmonary Exacerbations in CF	NCT02043717 Hadassah Medical Organization	2014–2015	CF
Prospective Intervention Study on Vitamin D in Patients With Cystic Fibrosis (D-vitamin)	NCT01321905 Karolinska Institutet	2010–2011	CF
Effects of Vitamin D Supplementation on Lung Function in an Acute Pulmonary Exacerbation of Cystic Fibrosis	NCT00788138 Emory University	2008–2010	CF
Vitamin D for Enhancing the Immune System in Cystic Fibrosis (DISC Study) (DISC)	NCT01426256 Emory University	2011–2017	CF
PK/PD of Vitamin D3 in Adults With CF	NCT03734744 University of Southern California	2019–2022	CF
The Role of Vitamin D3 in Pediatric Bronchiectasis Severity	NCT04411901 Heba Omara, Ain Shams University	2018–2019	CF
Efficacy of Intensive Cholecalciferol Monitoring and Supplementation on Serum vit D Levels in Pediatrics Patients With CF	NCT05276960 Hospital Infantil de Mexico Federico Gomez	2022–2023	CF
Vitamin D and Microbiota in Cystic Fibrosis	NCT02589444 Emory University	2015–2017	CF
Cholecalciferol for Vitamin D in Adult Cystic Fibrosis (CF) Patients	NCT00685971s Unity Health Toronto	2008–2013	CF

Note: Source: clinicaltrials.gov repository.

**Table 2:** Clinical trials in CF in the vitamin D category.

Title, ID, Sponsor, Year	Participant Flow	Adverse Events	Results
Improving Vitamin D Status In Cystic Fibrosis NCT00450073 Atlanta VA Medical Centre 2006–2011	Vit D3 Group: 9 completed, 1 not completed (10 started) Vit D2 Group: 10 completed Sunlamp group: 9 completed, 1 not completed (10 started)	NONE	25-hydroxyvitamin D Mean after 12 weeks (SD) [ng/mL] Vit D3 50,000 IU weekly Group: 21.2 (10.3) Vit D2 50,000 IU weekly Group: 24.4 (10.2) Sunlamp 5× weekly Group: 28.2 (3.2)
Vitamin D and Prebiotics for Intestinal Health in Cystic Fibrosis NCT04118010 Emory University 2020–2022	Vitamin D3 and Inulin Group: 7 completed, 3 not completed (10 started) Vitamin D3 and Placebo Inulin Group: 8 completed, 2 not completed (10 started) Placebo Vitamin D3 and Inulin Group: 7 completed, 3 not completed (10 started) Placebo Vitamin D3 and Placebo Inulin Group: 9 completed, 1 not completed (10 started)	NONE	Shannon Index Mean after 12 weeks (SD) Vitamin D3 50,000 IU weekly and Inulin 12 g daily Group Baseline: 5.02 (0.392) 12 Weeks: 5.02 (0.392) Vitamin D3 50,000 IU and Placebo Inulin Group: Baseline: 4.96 (0.165) 12 Weeks: 5.05 (0.169) Placebo Vitamin D3 and 12 g daily Inulin Group: Baseline: 5.28 (0.196) 12 Weeks: 5.24 (0.229) Placebo Vitamin D3 and Placebo Inulin Group: Baseline: 5.73 (0.081) 12 Weeks: 5.41 (0.097)
Open-label Vitamin D Trial for Patients With Cystic Fibrosis and Allergic Bronchopulmonary Aspergillosis NCT01222273 University of Pittsburgh 2010–2013	Cholecalciferol 4000 IU daily: 7 completed	NONE	Number of Participants With Aspergillus Induced IL-13 Responses in CD4+ T-cells after 6 Months 7 Change in Patient Total IgE Levels Mean after 6 Months (SD) [IU/mL] Baseline: 344.6 (284.9) 6 Months: 312.6 (77.66) Change in Patient Aspergillus Specific IgE Levels after 6 Months Baseline: 18.4 (14.7) 6 Months: 11.73 (3.581)
Clearance of 25-hydroxyvitamin D in Cystic Fibrosis (CF) NCT03104855 University of Washington 2017–2023	CF Group: 5 completed Healthy Group: 5 completed	Pain or sensation in the arm during infusion: 2/5 (CF Group) Bruising after blood draw: 1/5 (Healthy Group)	Metabolic Clearance of D6-25(OH)D3 (calculated as the administered dose of 25(OH)D3 divided by the area under the plasma concentration-time curve (AUC) CF Group 8 Weeks: 397 (73) Healthy Group 8 Weeks: 342 (41) AUC of D6-25(OH)D3 CF Group 8 Weeks: 58.3 (9.7) Healthy Group 8 Weeks: 67.2 (8.3) Terminal Half-life of D6-25(OH)D3 (ln2/k, where k is the slope of the terminal regression line estimated using ≥3 plasma concentrations) CF Group 8 Weeks: 16.2 (3.3) Healthy Group 8 Weeks: 15.8 (1.6) Volume of Distribution of D6-25(OH)D3 (in the central compartment is calculated as dose/C0, where the dose is the administered dose of 25(OH)D3 and C0 is the initial (estimated) concentration of drug in plasma) CF Group 8 Weeks: 8.4 (1.4) Healthy Group 8 Weeks: 7.2 (1.1) Metabolic Formation Clearance of D6-25(OH)D3 Metabolites (calculated as the daughter metabolite plasma AUC divided by the AUC of D6-25(OH)D3 (metabolite/parent AUC ratio)) CF Group 8 Weeks: 0.10 (0.02) Healthy Group 8 Weeks: 0.08 (0.01) Change in Serum Concentration of Calcium, Creatinine [mg/dL] AST, ALT [Units/L] CF Group 7 Days vs. Baseline: Calcium: 0.00 (0.37) Creatinine: 0.00 (0.03) AST: 0.60 (5.03) ALT: 0.00 (0.00) Healthy Group 7 Days vs. Baseline: Calcium: 0.14 (0.27) Creatinine: 0.00 (0.07) AST: −1.00 (2.55) ALT: 0.00 (0.00)
Safety, Efficacy, and Feasibility of High-dose Cholecalciferol in Paediatric Patients With Cystic Fibrosis NCT02613884 Johns Hopkins All Children’s Hospital 2016–2019	Treatment with High-Dose D3 250,000 IU once: 24 completed, 2 not completed (1 death, 1 loss to follow-up) (26 started)	ALL-CAUSE MORTALITY: 1 (3.85%) SERIOUS EVENTS: Lung infection: 1 (3.85%) Decreased lung function and weight loss: 2 (7.69%) Bronchopneumonia exacerbation: 1 (3.85%) Diarrhea: 3 (11.54%) Stomach ache: 3 (11.54%) Increased burping: 2 (7.69%) Nausea: 2 (7.69%) Heartburn/Reflux: 2 (7.69%) Constipation: 1 (3.85%) Elevated P: 4 (15.38%) Elevated PTH: 1 (3.85%) Elevated Ca: 1 (3.85%) Lung infection: 2 (7.69%) Hypoxemia: 1 (3.85%) Sinus infection: 1 (3.85%) Increased cough: 1 (3.85%)	Safety of a Single High-dose of Oral Cholecalciferol to Treat a Vitamin D Deficiency in Children With Cystic Fibrosis (serum calcium measurement after administration of treatment; treatment will be considered to be safe if the serum calcium level does not exceed 14 mg/dL14 mg/dL) Mean (SD) [mg/dL] Serum Calcium 1 Week: 9.57 (0.32) Serum Calcium 3 Months: 9.47 (0.41) Efficacy of a Single High-dose of Oral Cholecalciferol in Treating a Vitamin D Insufficiency/Deficiency in Children With Cystic Fibrosis (25OHD level measured after treatment at 3 months, 6 months, and 12 months; the treatment will be considered to be efficacious if the 25OHD level is greater than or equal to 30 ng/dL) Mean (SD/IQR) [ng/dL] Baseline: 22.69 (4.75) 3 Months: 26 (24 to 31) 6 Months: 30 (24 to 32) 12 Months: 27 (22.5 to 30.2) Feasibility of undertaking a large-scale randomized trial (acceptability and timing of previous outcome measures and obtain estimates to design a full-scale randomized trial by using both the efficacy measurement and the safety measurement; feasibility will be measured using a 5-item questionnaire that will be administered via telephone 1 week after administration of treatment) Number of Yes answers (%) Increased amount of nausea: 2 7.7% Increased frequency of emesis: 0 0.0% Increased amount of diarrhoea: 3 11.5% Constipation: 1 3.8% Increased gas production, such as burping or passing gas: 2 7.7% Increased amount of abdominal pain/stomach aches: 3 11.5% Increase in heartburn or reflux: 2 7.7% Number of No answers (%) Easy to take: 0 0.0% Something you would do next year if you had another low vitamin D level: 0 0.0% Prefer taking a one-time dose of vitamin D instead of a daily vitamin D: 0 0.0%

Note: Source: clinicaltrials.gov repository, own elaboration.

## Data Availability

No new data were created.

## Abbreviations

The following abbreviations are used in this manuscript:

ACE	Angiotensin-Converting Enzyme
AIH	Autoimmune Hepatitis
CF	Cystic Fibrosis
CFTR	Cystic Fibrosis Transmembrane Conductance Regulator
CPG	Clinical Practice Guidelines
CRP	C-Reactive Protein
CTLA-4	Cytotoxic T-Lymphocyte-Associated Protein 4
CYP	Cytochrome P450
DBP	Vitamin D Binding Protein
DM1	Diabetes Mellitus Type 1
EB	Epidermolysis Bullosa
EU	European Union
FEV1	Forced Expiratory Volume in 1 Second
FVC	Forced Vital Capacity
HAART	Highly Active Antiretroviral Therapy
IL	Interleukin
INF	Interferon
JEB	Junctional Epidermolysis Bullosa
MHCII	Major Histocompatibility Complex Class II
MS	Multiple Sclerosis
NKT	Natural Killer T-Cells
PBC	Primary Biliary Cirrhosis
PSC	Primary Sclerosing Cholangitis
QoL	Quality of Life
RD	Rare Disease
RDEB	Recessive Dystrophic Epidermolysis Bullosa
RXR	Retinoid X Receptor
SLE	Systemic Lupus Erythematosus
SSRI	Selective Serotonin Reuptake Inhibitor
TB	Tuberculosis
TLR	Toll-like Receptor
TNF	Tumor Necrosis Factor
UVB	Ultraviolet B
VDR	Vitamin D Receptor
1,25(OH)2D3	1α,25-dihydroxycholecalciferol (Calcitriol)
25(OH)D	25-hydroxycholecalciferol (Calcifediol)
